# Juxtaposition of heterochromatic and euchromatic regions by chromosomal translocation mediates a heterochromatic long-range position effect associated with a severe neurological phenotype

**DOI:** 10.1186/1755-8166-5-16

**Published:** 2012-04-04

**Authors:** Palma Finelli, Silvia Maria Sirchia, Maura Masciadri, Milena Crippa, Maria Paola Recalcati, Daniela Rusconi, Daniela Giardino, Laura Monti, Francesca Cogliati, Francesca Faravelli, Federica Natacci, Leonardo Zoccante, Bernardo Dalla Bernardina, Silvia Russo, Lidia Larizza

**Affiliations:** 1 Laboratory of Medical Cytogenetics and Molecular Genetics, Istituto Auxologico Italiano, Cusano Milanino 20095, Italy; 2 Dept. of Biology and Genetics for Medical Sciences, Università degli Studi di Milano, Milano 20133, Italy; 3 Dept. of Medicine, Surgery and Dentistry, Università degli Studi di Milano, Milano 20142, Italy; 4 Pathology Unit, Fondazione IRCCS, Ca' Granda, Ospedale Maggiore Policlinico, Milano 20122, Italy; 5 Department of Human Genetics,Ospedale Galliera, Genova 16128, Italy; 6 Clinical Genetics Unit, Department of Obstetrics and Pediatrics, Fondazione IRCCS, Ca' Granda, Ospedale Maggiore Policlinico, Milan 20122, Italy; 7 Child Neuropsychiatry Unit, Department of Life Science and Reproduction G.B. Rossi Hospital, Università di Verona, Verona 37126, Italy

**Keywords:** Balanced translocation, Heterochromatin, Position effect, Gene expression perturbation, Epigenetic modification

## Abstract

**Background:**

The term "position effect" is used when the expression of a gene is deleteriously affected by an alteration in its chromosomal environment even though the integrity of the protein coding sequences is maintained. We describe a patient affected by epilepsy and severe neurodevelopment delay carrying a balanced translocation t(15;16)(p11.2;q12.1)dn that we assume caused a position effect as a result of the accidental juxtaposition of heterochromatin in the euchromatic region.

**Results:**

FISH mapped the translocation breakpoints (bkps) to 15p11.2 within satellite III and the 16q12.1 euchromatic band within the *ITFG1 *gene. The expression of the genes located on both sides of the translocation were tested by means of real-time PCR and three, all located on der(16), were found to be variously perturbed: the euchromatic gene *NETO2/BTCL2 *was silenced, whereas *VPS35 *and *SHCBP1*, located within the major heterochromatic block of chromosome 16q11.2, were over-expressed. Pyrosequencing and chromatin immunoprecipitation of *NETO2/BTCL2 *and *VPS35 *confirmed the expression findings. Interphase FISH analysis showed that der(16) localised to regions occupied by the beta satellite heterochromatic blocks more frequently than der(15).

**Conclusions:**

To the best of our knowledge, this is the first report of a heterochromatic position effect in humans caused by the juxtaposition of euchromatin/heterochromatin as a result of chromosomal rearrangement. The overall results are fully in keeping with the observations in *Drosophila *and suggest the occurrence of a human heterochromatin position effect associated with the nuclear repositioning of the der(16) and its causative role in the patient's syndromic phenotype.

## Background

Patients with syndromic clinical phenotypes include an interesting subset of carriers of *de novo *balanced chromosomal rearrangements with no apparent loss or gain of genetic material. These abnormalities can be explained by the loss of function of a dose-sensitive gene disrupted by one rearrangement breakpoint [1-3]. The breakpoints of the balanced chromosomal rearrangements associated with a clinical phenotype are often molecularly mapped in an attempt to identify the disease-causing genes affected by the abnormality. Although the rearrangements may lead to the direct disruption of one or two genes, this is not always the case. It has also been shown that breakpoints occur outside the genes themselves and affect their regulation by causing a change in their position within the genome, a phenomenon known as the "position effect" (PE). Investigations of the PE have required intensive experimental effort because of the unique features of each case and the often scarcely defined length of the involved genomic region.

A number of potential mechanisms can be suggested to explain the position effects of chromosomal rearrangements in humans for a review see ref. [4]: 1) the separation of a gene from its enhancer, promoter or locus control region; 2) juxtaposition to an enhancer element from another gene; 3) the removal of the long-range insulator or boundary elements; 4) competition with another enhancer; 5) alterations in local chromatin structure; 6) alterations in nuclear organisation [5,6]; and 7) position effect variegation (PEV), when the genes are moved into close proximity to constitutive heterochromatin and their activity become unstable and leads to variegated patches of gene expression.

PEV was first observed in *Drosophila *and was so named because of the variegated pattern of expression of euchromatic genes transposed to locations near to pericentric heterochromatin as a result of natural or induced genetic rearrangements [7-10]. The chromosomal position effect can spread over distances of 1 Mb or more, and generally reflects a gradient of gene inactivation that is inversely correlated with distance [10], although it can also be affected by the local context of a gene [11].

Some genes are specifically adapted to be expressed exclusively in a heterochromatic context (heterochromatic genes) [9]. They are also influenced by PEV, but their behaviour is opposite to that of genes located in euchromatic chromosomal regions (euchromatic genes). The regulatory implication of genes residing in heterochromatic regions was first evidenced in a pioneering study that linked PEV to euchromatic/heterochromatic rearrangements: the variegation of the *light *gene, known as *Dmel\lt*, encoding a protein containing a zinc ion binding domain and residing at the heterochromatin side of the rearrangement breakpoint, was enhanced by an increased dose of Y chromosome constitutive heterochromatin, whereas the variegation of genes located on the euchromatic side of the breakpoint was suppressed [12]. Subsequently, it was found that a number of other heterochromatic genes show similar heterochromatic dependence [13-15], some of which are unique in terms of function and protein coding [16].

Sequencing projects have led to the discovery of hundreds of heterochromatic genes in Drosophila, plants and mammals, but no variegation effects have yet been reported in humans, although they have been observed in transgenic mice bearing incomplete functional gene domains that became inserted into heterochromatic regions [17].

Given the variability of chromosomal rearrangements, rare patients carrying unique chromosomal abnormalities often show single or multiple clinical signs that cannot be assigned to any recognisable syndrome. We here describe the molecular cytogenetic, genetic and epigenetic characterisation of a "private" balanced chromosomal translocation t(15;16)(p11.2;q12.1)dn carried by a patient affected by epilepsy and severe neurodevelopment delay. Fluorescence in situ hybridisation (FISH) breakpoint mapping and analyses of the quantitative expression and promoter epigenetic signatures of the genes at or near the breakpoints showed that a simple chromosomal rearrangement may involve multiple genes as a result of a long-range heterochromatic position effect.

## Results

### Clinical presentation

The proband is the first and only daughter of healthy and unrelated parents, and was born in the 39^th ^g.w. after an uncomplicated pregnancy with a birthweight of 3480 g (50^th ^centile), a length of 50 cm (50^th ^centile), and a head circumference of 33.5 cm (10^th ^centile). At birth, she was affected by neonatal respiratory distress and hypotonia.

During her first day of life, she experienced brief partial seizures with secondary generalisation that were treated with phenobarbital (PB) but recurred several times a week until she was two months old, when they started appearing as clusters of infantile axial spasms. The spasms disappeared after treatment with valproic acid (VPA), and the baby started to smile and gained control of her head. At the age of six months, the spasms returned and became daily until she was 30 months old, despite the administration of adrenocorticotropin hormone (ACTH) and gamma-vinyl GABA (GVG). Interictal electroencephalography (EEG) revealed the presence of frequent paroxysms involving the temporo-parietal regions of both hemispheres, with left predominance in the absence of a hypsarrhythmic pattern.

Subsequently, the spasms became progressively less frequent with VPA treatment, occurring only a few times a month during sleep or upon awakening. They finally disappeared when the patient was five years old, and interictal EEG revealed normal findings during wakefulness and rare focal spikes on the parietal regions only during sleep.

From the age of four years, the patient's clinical picture progressively improved. Now aged 12 years, she can sit alone, has hypotonic-dystonic cerebral palsy, is averbal, and suffers from very severe cognitive impairment, but her relationships are relatively good.

Cerebral MRI at the age of three years revealed a left temporo-parietal focal abnormality suggesting a prenatal insult with micropolygyria.

A physical examination at the age of 10 years showed a length of 120 cm (< 3^rd ^centile), a weight of 20 kg (< 3^rd ^centile), and a head circumference of 49 cm (< 3^rd ^centile), together with brachyplagiocephaly, wide palpebral fissures with mild lateral ectropion and long eyelashes, thin fingers, and severe thoracic scoliosis.

The patient was originally referred to our laboratory because of suspected Angelman syndrome, which was ruled out as *SNRPN *methylation analysis showed a biparental pattern and no *UBE3A *gene mutation was found. Rett syndrome was ruled out by means of mutation screening of the *MeCp2 *and *CDKL5 *genes.

### Translocation breakpoints analysis

Karyotype analysis showed a balanced translocation t(15;16)(p11.2;q12.1)dn.

**Figure 1:**
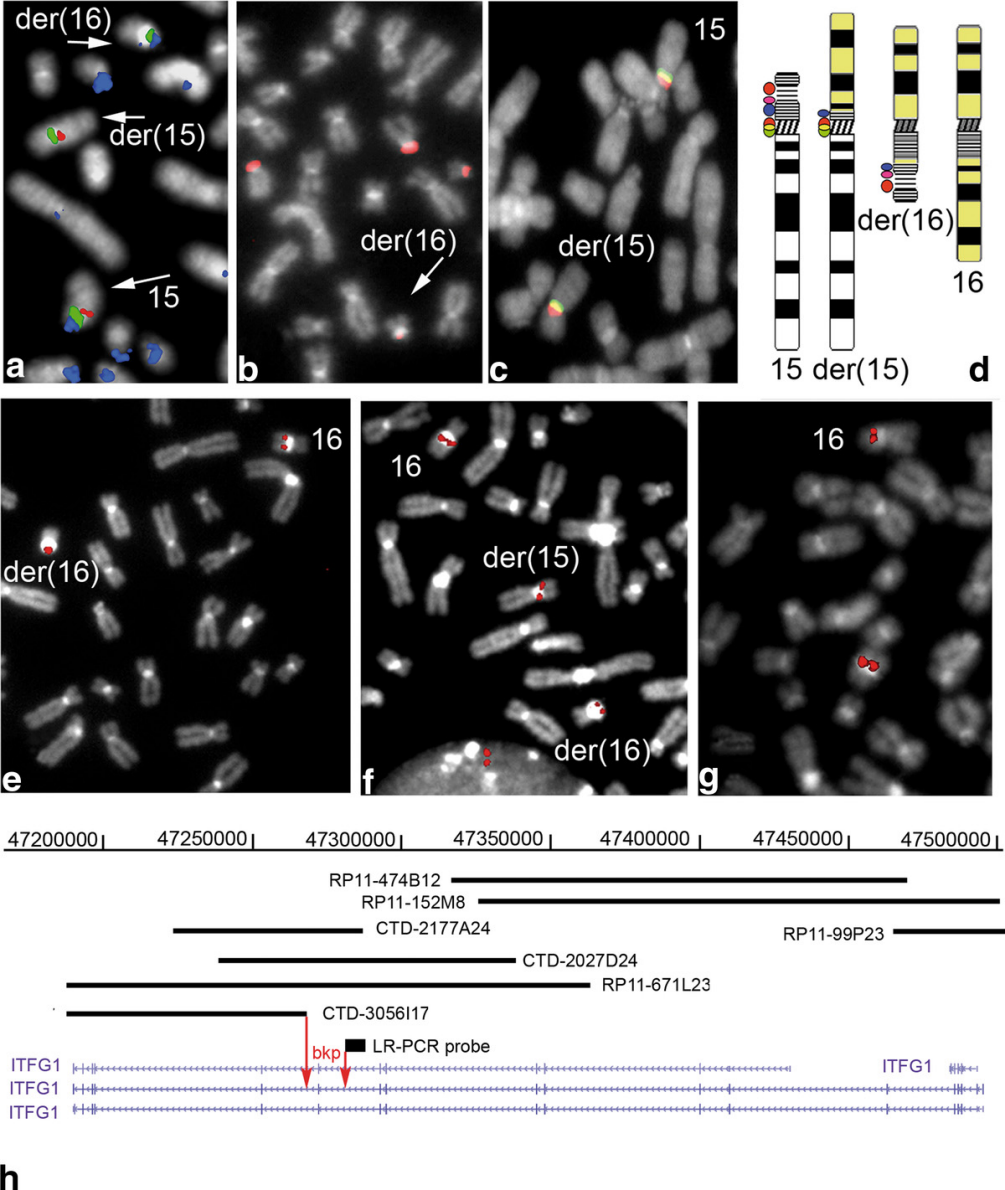
**FISH mapping of the 15p bkp (A, B, C, D)**. **A) **The D15Z1 satellite III probe targeting the 15p bkp gave a signal (green) on both derivative chromosomes 15 and 16 (arrowed); the beta satellite probe (RPC1-98 C19) gave a signal (blue) only on der(16) (arrowed); and the GABRB3 probe targeted both 15q chromosomes (red). **B) **The rDNA probe gave a signal on der(16). **C) **FISH of α-satellite D15Z3 (green) and D15Z4 (red) probes. **D) **Ideograms showing schematic FISH results concerning both derivative chromosomes of t(15;16) in comparison with normal chromosomes 15 (left) and 16 (in yellow on right). The red circle represents the NOR probe signals, the purple circle the beta satellite signals, the blue circle the satellite III signals, and the red and green circles (merged to yellow) the alpha satellite probe signals. FISH characterisation of the 16q bkp (E, F, G, H). **E) **The CTD-3056I17 probe only gave a signal on der(16); **F) **The CTD-2027D24 probe gave a signal on both derivative chromosomes; **G) **The long-range PCR probe gave a signal only on der(16); **H) **Physical map of chromosome 16q containing the bkp: the red arrows point to the bkp region. The scale refers to the February 2009 human draft sequence (hg19) from the UCSC genome browser.

FISH characterisation narrowed the 15p breakpoint region to within 15p11.2 satellite III (D15Z1) on the basis of the fact that probe D15Z1 gave a signal on both derivative chromosomes 15 and 16 (Figures 1A and 1D), whereas RPC1-98 C19 and RPC1-21I10 (containing beta satellite sequences) only gave a signal on der(16) (Figures 1A and 1D), as did the rDNA probe RPC5-1174A5 (Figures 1B and 1D). As expected, the α-satellite probes D15Z3 and D15Z4 gave a signal on the centromere of der(15) (Figures 1C and 1D).

The 16q translocation bkp was mapped to 16q12.1 using a BAC contig and was localised within the *ITFG1 *gene, which is disrupted: the contiguous BACs showed different hybridisation patterns, with CTD-3056I17 only giving a signal on der(16) (Figure 1E), CTD-2027D24 giving a signal on both derivative chromosomes (Figure 1F), and RP11-474B12 giving a signal only on der(15) (data not shown). In order to refine the localisation of the 16q breakpoint, a 7 kb long-range PCR genomic fragment, covering the distal part of *IVS12*, was hybridised with patient metaphases. This probe only gave a signal on der(16), the intensity of which was comparable with that of the normal chromosome 16, thus allowing the bkp region to be approximately narrowed to a 12 kb interval (chr16:47268711-47280772, hg19) (Figure 1G and 1H). All of the probes and the FISH results are shown in Additional file 1: Table S1.

In order to investigate whether the translocation involving the 15p heterochromatic blocks favoured a re-localisation of the 16q euchromatic bkp regions in a heterochromatic environment, we performed tripled i-FISH experiments using patient lymphoblast cells derived from three different cultures and hybridised with probes D15Z1, GABRB3 and RPC1-98 C19. For each experiment, at least 100 nuclei were analysed by evaluating the co-localisation of the 16q bkps with regions recognised by the probe RPC1-98 C19, which contains beta-satellite DNA.

**Figure 2:**
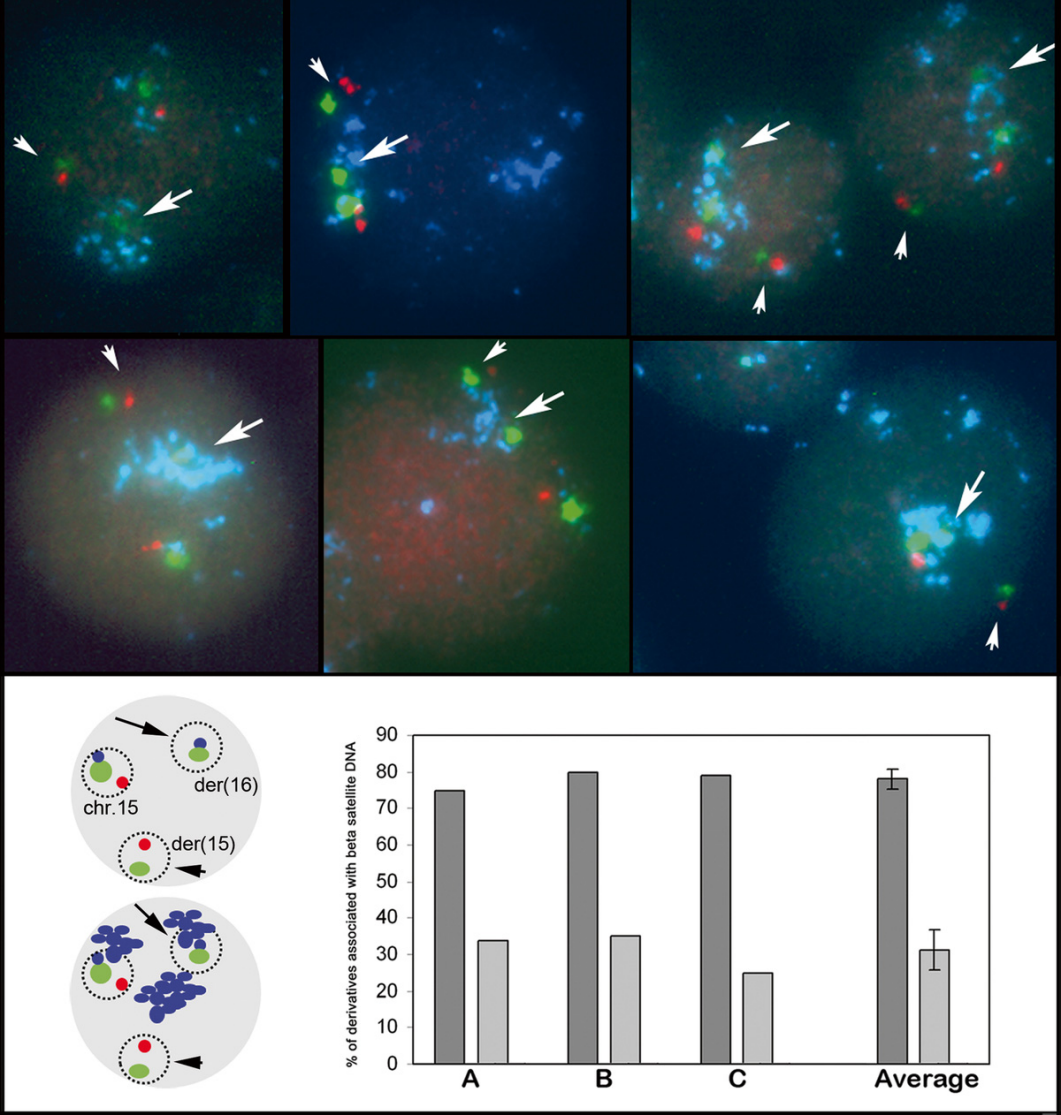
**Nuclear position of derivative chromosomes with beta satellite regions**. i-FISH (top) performed using D15Z1 (green), GABRB3 (red) and RPC1-98 C19 (blue) containing beta-satellite DNA. The large and small arrows respectively indicate der(16) and der(15). Chromosome 15 is not shown but it occupies the region in which all three hybridization signals are located, with the green signal larger than those identified on the two derivatives. At the bottom left a drawing showing the expected hybridization pattern allowing to recognize the three different chromosomes (der (16), der (15) and chromosome 15) on nucleus. The picture above shows only the expected pattern of hybridization on the three chromosomes in the drawing below is also shown acrocentric regions recognized by the probe containing beta-satellite DNA. At the bottom right graphs A, B, and C show the rate of co-localisation of der(16) (dark grey) and der(15) (light grey) to the regions targeted by the beta satellite. The results average of three experiments is shown on the right.

We observed der(16) co-localisation with heterochromatic regions more frequently than der(15) co-localisation in all of the experiments (Figure 2).

### Array CGH analysis

The analysis excluded the gain or loss of any genomic region encompassing the der(15)/der(16) breakpoints and flanking regions, including the loci monitored for gene expression. It also revealed a loss on chromosome 9 and a gain on the X chromosome, neither of which were listed in the reference Database of Genomic Variants http://projects.tcag.ca/variation, a catalogue of structural variations identified in healthy subjects. In detail, high-resolution a-CGH analysis identified a paternal deletion of at least 28 kb at 9p24.1 (chr9:6,215,819-6,244,723, hg19) and a maternal duplication of at least 500 kb at Xp22.2 (chrX:11,686,237-12,187,337, hg19) (Additional file 2: Figure S1). Two genes, *MSL3 *and *RFMPD4*/*PDZD10 *map to Xp22.2, the latter covering a duplication bkp and hence being hypothetically interrupted. The 9p deletion disrupts the *IL33 *gene.

### XCI pattern

In order to verify whether the maternally derived CNV localised on chromosome X could be involved in the patient's phenotype, X chromosome inactivation assay was performed analysing AR and DXS6673E loci. While the AR locus was uninformative, the DXS6673e locus displayed a random X inactivation in the mother (59:41) whereas the patient showed a preferential X inactivation of the maternal allele (82:18; Additional file 3: Figure S2). These results indicate no role of this CNV in the patient phenotype.

### Gene expression

**Figure 3:**
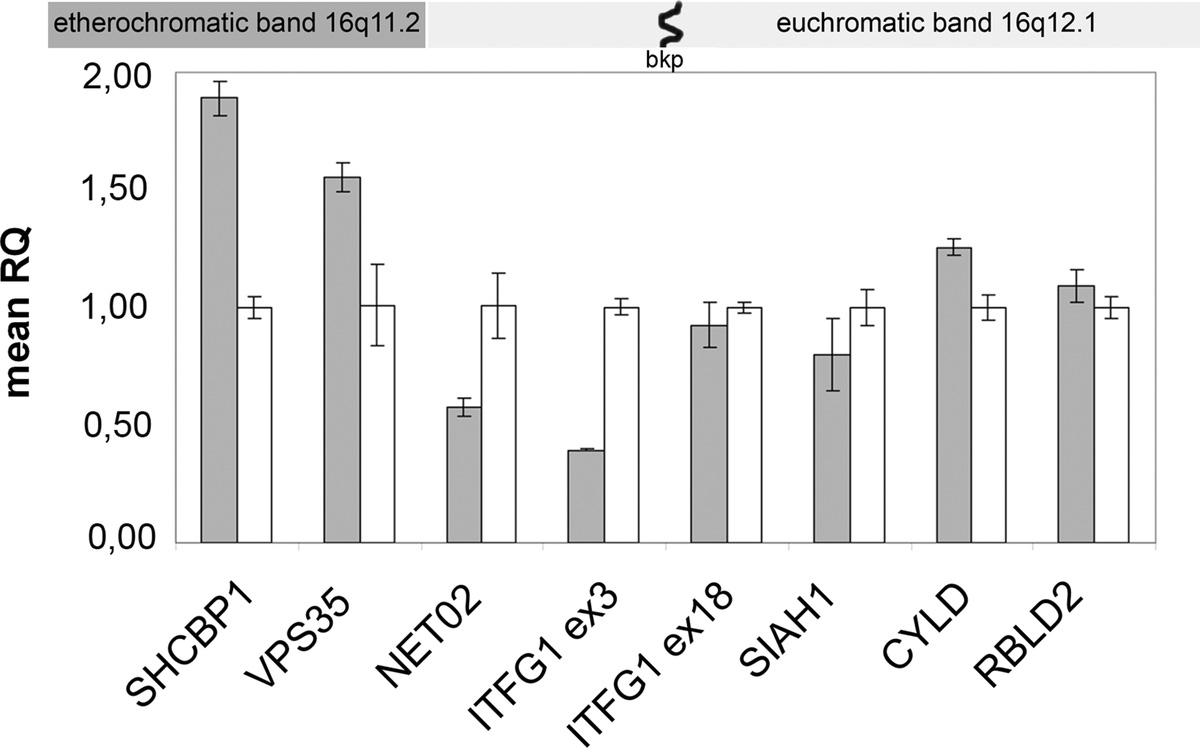
**Real-time quantitative PCR showing the relative quantification (RQ) of *ITFG1 *cDNA amplification product obtained using ex-18 primers, which was found to be half that obtained using ex-3 primers**. The expected ΔΔCt ratio is ≅1 when both alleles are expressed, and 0.5 when only one allele is expressed. The genes are indicated on the abscissa and the average of three recorded expression levels on the ordinate. The grey and white bars respectively represent the case and control.

Real-time PCR using primers positioned on the opposite sides of the bkp within *ITFG1 IVS12 *asses that the amount of the 5' portion of the transcript was comparable with that of the control and half that of its 3' portion, thus confirming the FISH results (Figure 3).

Given the complex syndromic phenotype of the patient, we tested whether the breakage and juxtaposition of 15p constitutive heterochromatin to the 16q12.1 euchromatic region disturbed the expression of the intact genes flanking the 16q bkp. We first analysed four genes whose expression profiles and functions suggested a possible causative role: *VPS35 *and *NETO2/BTCL2*, which respectively map to 16q11.2 heterochromatic and 16q12.1 euchromatic bands, and are localised 0.5 Mb and 62 kb from the bkp on der(16), and *SIAH1 *and *CBLN1*, which both map to 16q12.1 and are localised 1.1 Mb and 2 Mb from the bkp on der(15). Real-time PCR revealed halved expression of *NETO2/BTCL2 *and the over-expression of *VPS35*, both located on der(16), whereas the expression of SIAH1 was comparable with that of the control (Figure 3). The expression of the brain-specific *CBLN1 *gene was not quantifiable because it was low in all of lymphoblast cell lines.

In order to establish whether other more distant genes were transcriptionally perturbed, we also analysed the expression of the der(16) genes lying at distances from bkp of 1 Mb (*SHCBP1*), 0.56 Mb *(ORC6L)*, 0.67 Mb (*GPT2*) and 0.27 Mb (*DNA JA2*); *SHCBP1 *and *ORC6L *are located in the major heterochromatic block of chromosome 16 (16q11.2), and *GPT2 *and *DNA JA2 *in the euchromatic band 16q12.1. We also monitored the expression of the following genes located on der(15) at 16q12.1: *ABCC12 *(0.9 Mb from the bkp), *N4BP1 *(1.4 Mb), *ADCY7 *(3 Mb), *BRD7 *(3.1 Mb), *CYLD *(3.5 Mb), *SALL1 *(3.9 Mb) *RBL2 *(6.2 Mb), *HERPUD *(9.7 Mb) and *CKLF *(19 Mb). Only the *SHCBP1 *gene was expressed at higher levels than the control levels (Figure 3), whereas the expression of most of the genes (*DNA JA2, ADCY7, BRD7, CYLD *and *RBL2*) was comparable with that of the controls (data non shown). In relation to the remaining genes (*ORC6L, ABCC12, N4BP1, SALL1, HERPUD *and *CKLF*), we could not draw any conclusions because of their low level of expression in the lymphoblast cell lines or the variability of expression in the control samples (*GPT2*).

### Epigenetic characterisation of the promoter regions

It is well known that H3mK9, H3mK27 and CpG site methylation occurs in silenced gene promoters, whereas the acetylation of histones H3 and H4 and H3 lysine 4 methylation correlate with transcriptionally active genes. In order to assess the epigenetic status of the intact genes flanking both sides of the 16q breakpoint, lymphoblastoid cell lines from the patient and controls were investigated for their DNA methylation status and chromatin modifications in the regulatory regions of the genes whose expression was disturbed or slightly perturbed. In particular, we tested the promoter regions of the *NETO2/BTCL2, SIAH1, CYLD *and *RBL2 *genes.

**Figure 4:**
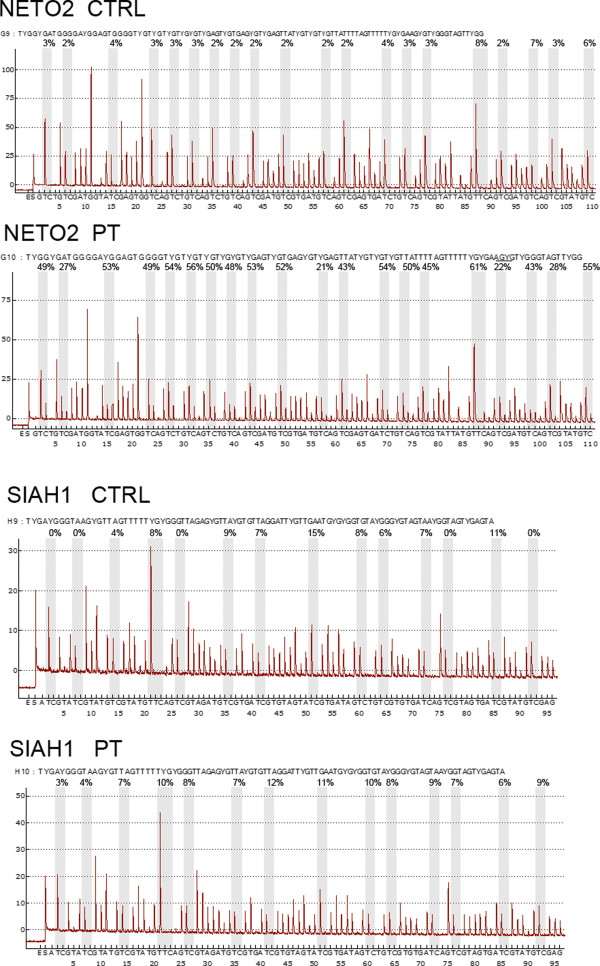
**Pyrograms indicating the percentage of methylation of positions 20 CpG and 14 CpG within the *NETO2 *and *SIAH1 *promoter regions in the patient (PZ) and normal lymphoblast cell lines (CTRL)**. The mean methylation percentages are shown in brackets.

The promoter methylation analysis was made using a pyrosequencing assay that quantitatively measures the methylation of all CpG sites. In the normal samples, *NETO2/BTCL2, SIAH1, CYLD *and *RBL2 *were demethylated at all of the analysed CpG sites; in the patient, and in line with the gene expression results, we found a *NETO2 *promoter methylation pattern indicating the epigenetic silencing of one allele (mean 50% methylation) (Figure 4), whereas all of the other gene promoters located on der(15) (*SIAH1, CYLD *and *RBL2*) showed a methylation pattern comparable with that of the normal samples (Figure 4).

**Figure 5:**
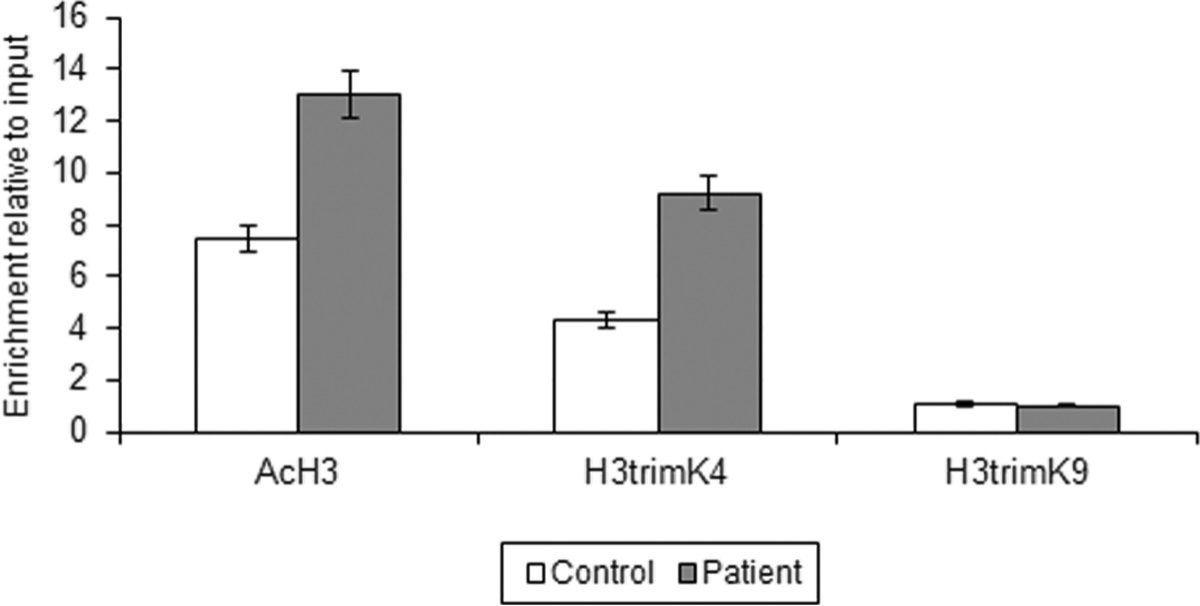
**Real-time quantitative PCR of the immunoprecipitated chromatin *VPS35 *promoter**. The results of two independent experiments.

We then tested seven different modifications on the histone tails: H3, H4 acetylation, and H3 di- and tri-methylation on lysine 4 (associated with an active chromatin state), and H3 di- and tri-methylation on lysine 9 and H3 di-methylation on lysine 27, which are specific for transcriptionally silenced chromatin. The ChIP assays showed H3 K9 di- and tri-methylation and H3 K27 di-methylation in the *NETO2/BTCL2 *promoter (Additional file 4: Table S2), whereas *VPS35 *and *SIAH1 *showed the same qualitative histone code as the control. As *VPS35 *was up-regulated, we used Real-time PCR on chromatin immunoprecipitates to assess the quantitative changes in the epigenetic modifications related to the trascriptionally competent chromatin, and found increased H4 acetylation and H3 tri-methylation on lysine 4 (Figure 5).

## Discussion

We believe that this may be the first description of a human disease involving a heterochromatic PE, by means of which intact euchromatic and heterochromatic genes are respectively silenced or enhanced as a result of euchromatin/heterochromatin juxtaposition to constitutive heterochromatin. Although our molecular analysis could not assay variegation in the classical sense of PEV, at least three genes showed perturbed expression and behaviour equivalent to that of *Drosophila *genes in the same chromosomal context [8]. As in *Drosophila*, we found the over-expression of heterochromatic genes (*VPS35 *and *SHCBP1*) located at 16q11.2, and the under-expression of the euchromatic gene (*NETO2/BTCL2*) located at 16q12.1. In keeping with the expression results and the findings observed in *Drosophila *[18], the silenced *NETO2/BTCL2 *"euchromatic" gene on der(16) showed promoter DNA methylation and H3 K9 di- and tri-methylation/H3 K27 di-methylation histone modifications, whereas the over-expressed *VPS35 *"heterochromatic" gene showed an increase in H4 acetylation and H3 tri-methylation on lysine 4 in comparison with the control. These findings confirm that these chromatin modifications act as universal markers of active/inactive genes in both the heterochromatic and euchromatic bands [18]. We did not observe any epigenetic silencing of the assayed der(15) genes *SIAH1, CYLD *and *RBL2*.

The expression profiles and epigenetic findings consistently revealed the presence of an alteration only on one side of the translocation breakpoint, on the der(16) chromosome. Interestingly but reasonably expected, we also observed that der(16), owning chromosome 15 satellites, is more frequently associated with acrocentric chromosomes beta satellite heterochromatic blocks than der(15). In line with the results of recent studies leading to the conclusion that the reorganisation of chromosomal territories may be an important step in the process of gene silencing [6], our overall findings suggest that the different behaviour of the two derivative chromosomes may be caused by the different nuclear re-localisation of der(16).

Treatment with VPA, acting also as HDAC inhibitor, could modify the epigenetic profile of the patient [19,20], nevertheless the epigenetic signature observed in the derivative chromosomes is coherent with a PE mechanism and not altogether justifiable by the drug treatment.

In terms of genotype-phenotype correlations, our assumption is that most of the observed clinical signs of our patient can be attributed to the *de novo *balanced translocation. The clinical phenotype of the patient can be interpreted as an effect of the disruption of the *ITFG1 *gene combined with the heterochromatic PE effect (assuming that the genes in question are dose sensitive).

The 16q translocation bkp disrupts the *ITFG1 *gene (an inhibitory regulator of PP2A that plays a role in modulating kinase levels in the ATM/ATR signalling pathway), leading to a truncated transcript that is not degraded by nonsense-mediated RNA decay [21]. Moreover, some of the perturbed genes can be plausibly considered causative candidates on the basis of their expression profiles and functions. These include *NETO2/BTCL2*, which is highly expressed in fetal brain and may play a role in the development and/or maintenance of neuronal circuitry [22,23] as a similar gene in rats encodes a protein that modulates glutamate signalling in the brain by regulating kainate receptor function [24,25]. The over-expressed *VPS35 *is also highly expressed in the brain, and encodes an essential component of the retromer complex [26,27]. The investigation of other plausible candidates (e.g. *CBLN1*) was precluded by their lack of expression in lymphoblastoid cell lines.

The imbalance in the X chromosome inherited from the patient mother could contribute to the patient's phenotype as it contains *MSL3 *gene and *RFMPD4/PDZD10 *gene, acting respectively as chromatin remodelling/transcription regulating factor [28,29] and as positive regulator of dendritic spine morphogenesis, with a role in the maintenance of excitatory synaptic transmission [30]

Nonetheless, the X inactivation analysis showed a random X inactivation in the mother and a preferential maternal X inactivation in the daughter, excluding a role for this CNV in the patient's phenotype.

## Conclusion

Our results, which were obtained from genetic and epigenetic studies, suggest the occurrence in human of the PE due to an euchromatin/constitutive heterochromatin juxtaposition. To our knowledge this is the first report of a human disease involving this kind of PE mechanism.

Despite our extensive characterisation, the multiple genes that may be involved prevent a full understanding of the pathogenetic mechanism. However, we can reasonably assume that the heterochromatic PE in our case leads to a situation that is comparable with that observed in contiguous gene syndromes. Other carriers of balanced translocations with a breakpoint in heterochromatic sequences need to be similarly studied in order to be able to generalise the contribution of heterochromatic PE to human pathology.

## Materials and methods

### Cytogenetic and FISH analyses

QFQ-banded metaphases prepared from peripheral blood lymphocytes using standard procedures were analysed, and their karyotype was described in accordance with *ISCN *(2009) [31]. The probes used for the FISH characterisation of the 15p bkp were the RPC1-98 C19 and RPC1-21I10 clones, provided by Invitrogene (cat. N. RPCI1.C), both containing beta-satellite DNA and targeting 15p11 and 15p13 heterochromatic bands [32]; the RP5-1174A5 clone, which contains the DNA/rRNA coding sequences (NOR regions) http://www.biologia.uniba.it/rmc/5-alfoidi/dJ1174A5.html has been selected from RPCI-5 PACs library provided by the YAC Screening Centre (Dibit, HSR, Milan, Italy); the D15Z1 satellite III probe mixed with the GABRB3 probe (Abbott, cat N. 05 J22-015); and the pMC15 (D15Z3) and pTRA25 (D15Z4) clones containing specific chromosome 15 alpha satellite sequences [33] were kindly provided by Prof. Mariano Rocchi, University of Bari. The contiguous BACs, belonging to the RPCI-11 and the CTD libraries and used for the FISH characterisation of the 16q bkp, were selected using the UCSC Genome Browser (http://genome.ucsc.edu/, hg19) and provided by Prof. Mariano Rocchi (RPCI-11 clones) and by Invitrogene (CITB Human D BAC clones, cat. N. 96012D) The 16p bkp was also characterised by means of a 7 kb probe covering part of *ITFG1 *intron 12, which was constructed by means of long-range PCR using the Expand 20KB^PLUS ^PCR System (Roche, cat N. 11811002001) as previously described [34]. The primer pair (fw 5-GGCACCATCTTGGCACACTGCAACCTC3- and rev 5-ATTAGGAACCAGGCCGCACGACAGGAG 3-) was designed from the genomic contig NT_010498, and the amplicon sequences (chr16:47,280,772-47,287,772, hg19) were checked by BLAT against the human sequence in order to ensure amplification specifity. Ten nanograms of clone CTD-2027D24 were used as a template. The 7 kb PCR product was run in 1% low-melting agarose and subsequently purified. All of the BAC/PAC probes were nick-translation labelled with biotin (Roche, cat N. 11093070910), digoxigenin (Roche, cat N. 11093088910) or CY3-dUTP (Amersham, cat N.PAS3022), whereas the PCR product probe was labelled by means of oligopriming (Prime-It Fluor Fluorescence labelling kit, Stratagene, cat. N. 300380). All of the FISH probes were used following the protocols of Lichter and Cremer with minor modifications [35]. Each of the BAC clones was previously tested on chromosomal spreads from normal controls, and only those giving a signal on the chromosomal band indicated by the Genome Browser (UCSC) were used. The specificity of the probe obtained by means of long-range PCR was validated by a FISH experiment that gave a single signal on chromosome 16q11.2. Digital images were obtained using a Leica DMR epifluorescence microscope (Leica Imaging Systems Ltd) equipped with a CCD camera (Cohu Inc). DEAC, FITC, Cy3, and DAPI fluorescence signals were detected using specific filters. The images were recorded, pseudocolored, and merged using QFISH software (Leica Imaging Systems Ltd), and finally edited using Adobe Photoshop CS4 (Adobe Systems).

### Array comparative genome hybridisation (CGH) analysis

For array CGH analysis genomic DNA was extracted from proband's and parent's whole blood using the Dneasy Blood & Tissue Kit (Sigma-Aldrich, cat. N. NA2020) according to the manufacturer's instructions. Pooled DNA from the peripheral blood of 10 healthy donors (Promega, cat. N. G1521), sex-matched to the samples, was used as a reference. Genome scan was performed by the Human Genome CGH Microarray Kit 244 K (Agilent Technologies, cat. N. G4411B). Briefly, 3 μg of DNA from the test and the normal reference were processed according to the manufacturer's protocol. Images were extracted using Agilent Feature Extraction software 9.1 and analysed by DNA Analytics 4.0 software. A log ratio plot between test and reference genomic DNA was assigned so that aberrations in test DNA copy number at a particular locus are observed as the deviation of the ratio plots from a modal value of 0. Aberration calls were identified by the ADM-2 algorithm.

### X inactivation analysis

The X-inactivation pattern was assessed using the Humara Androgen Receptor locus and the DXS6673E locus methylation assay as previously described [36,37]. PCR products were run by capillary electrophoresis and XCI values determined in heterozygous cases using the formula previously reported [38]. This formula calculates the XCI pattern as (d1/u1)/(d1/u1 + d2/u2), where d1 and d2 represent the two peak areas from the digested sample and u1 and u2 are the corresponding areas of the alleles obtained from undigested DNA.

### Expression analysis

Briefly, RNA was extracted from the patient and controls lymphoblast cell lines using Trizol (Gibco BRL. cat N. 15596-018) DNAse-treated (Invitrogene, cat N. 18068-015), and cDNA synthesized using the SupscriptIII Kit (Invitrogen, cat N. 117-52-050) protocol with gene-specific reverse strand primers.

The investigated genes were *SHCBP1, VPS35, ORC6L, GPT2, DNA JA2, NETO2/BTCL2, ITFG1, ABCC12, SIAH1, N4BP, CBLN1, ADCY7, BRD7, CYLD, SALL1, RBL2, HERPUD*, and *CKLF*. The primer pairs designed for each gene, which spanned coding sequences, are listed in Additional file 5: Table S3. Quantitative RT-PCR was performed using the comparative threshold cycle method of the Syber-Green (SYBR^®^Green PCR Master Mix - Applied Biosystem, cat N. 4309155) protocol, as described by Livak and Aarskog and Vedeler [39,40]. The RT-PCR and data analyses were carried out using an ABI PRISM 7900HT Sequence Detection System (Applied Biosystem). The estimated expression of all of the genes was computed from three independent retrotranscriptions obtained from three different RNA extractions; the threshold cycle number (Ct) values were obtained from three replicas, and normalised to the endogenous *HGPRT *control gene. RNA from three healthy human controls (one female and two males) were used as the calibrator sample.

Ct values were determined for all of the PCR reactions, and a comparative Ct method was used to calculate the copy numbers of the expressed transcripts. The relative quantity of expressed transcript was calculated as RQ = 2-(ΔΔCt ± standard deviation [SD]) [39,40].

### Methylation analysis

The methylation status of the *NETO2/BTCL2, SIAH1, RBL2 *and *CYLD *promoter regions was analysed by means of pyrosequencing technology. The bisulfite conversion of genomic DNA (1 μg) was obtained using the EZ DNA methylation kit (Zymo Research, cat N. D5002). After bisulfite treatment, PCR was carried out in a final volume of 50 μl with 2.5 units of Promega Go-Taq polymerase (Promega, cat N. M8301). The primers for modified sequences are summarised in Additional file 4: Table S3. The PCR conditions were 45 cycles of 95°C for 30 sec, 52°C for 30 sec, and 72°C for 30 sec, followed by 72°C for 5 min; 30 μl of the PCR product were used for the pyrosequencing assay carried out using the sequencing primers (Figure 3).

The pyrosequencing reactions were performed in a PSQ HS 96 System (Biotage, Uppsala, Sweden) by using Pyro Gold Reagent kits (Qiagene, cat. N. 972804). Methylation was quantified using Pyro Q-CpG Software (Biotage), which calculates the ratio of converted C's (T's) to unconverted C's at each CpG site and expresses this as a percentage of methylation. To assess the methylation pattern under normal conditions, we analysed a control lymphoblastoid cell line and different normal DNA from peripheral blood lymphocytes.

### Chromatin immunoprecipitation assay (ChIP)

Chromatin immunoprecipitation was assayed using the chromatin immunoprecipitation assay kit and anti-acetyl-histone H3 (AcH3) (Upstate Biotechnology, cat N. 06-599), anti-acetyl-histone H4 (AcH4) (Upstate Biotechnology, cat N. 06-866), anti-di and -trimethyl-histone H3 Lys4, H3DimK4 (Upstate Biotechnology, cat N. 07-030) and H3TrimK4 (Upstate Biotechnology, cat N. 04-745), anti-di and -trimethyl-histone H3Lys 9, H3DimK9 (Upstate Biotechnology, cat N. 07-441) and H3TrimK9 (Upstate Biotechnology, cat N. 07-442), and anti-dimethyl-histone H3 Lys27 (H3DimK27) (Abcam anti - H3 Trim K27 cat.N. ab6002) antibodies in accordance with the manufacturer's instructions, with minor modifications. Chromatin was immunoprecipitated from 2 × 10^6 ^lymphoblastoid cells (per antibody) taken from the patient and control [41].

Quantitative RT-PCR was performed on immunoprecipitated chromatin as previously described in order to verify the quantitative changes in histone modifications on the *VPS35 *promoter. The primer sequences specific for the *VPS35, NETO2/BTCL2, SIAH1, PGK1 *and *HPRT *genes (the last two used as controls) are shown in Additional file 6: Table S4.

## Consent

Written informed consent to the research investigation, which was approved by the Ethical Clinical Research Committee of Istituto Auxologico Italiano, was obtained from the proband's parents.

## Abbreviations

ACTH: Adrenocorticotropin hormone; aCGH: Array Comparative Genomic Hybridization; CNVs: Copy Number Variants; *ChIP*: Chromatin Iimmune Precipitation; EEG: Electroencephalography; FISH: Fluorescence in situ Hybridization; GVG: Gamma vinyl GABA(gamma aminobutyric acid); i-FISH: Interphase Fluorescence in situ Hybridization; MRI: Magnetic Resonance Imaging; NOR: Nucleolar Organization Region; PB: Phenobarbital; PE: Position Effect; PEV: Position Effect Variegation; QFQ: Q-banding methods using quinacrine; VPA: Valproic acid; UCSC: University of California Santa Cruz.

## Competing interests

The authors declare that they have no competing interests.

## Authors' contributions

PF designed and initiated the study, monitored data collection and analysis for the whole study. SMS, LL and SR contributed to the study design and in the interpretation of data. MPR, PF and DG did conventional cytogenetics and the FISH experiments and interpreted the data. SR and MM did the expression analyses and interpreted the data. MC and DR performed a-CGH analysis and i-FISH experiments and interpreted the data. SMS and LM did the ChIP and Pyrosequencing assays. FC did the SRNPN methylation analysis and the mutation screening of MeCp2 and CDKL5. LZ, B DB, NF, and FF performed clinical investigation at different times and reviewed all clinical records. PF drafted the manuscript. PF, SS and LL took part in critical revision of the manuscript. PF and LL did the final approval of the manuscript. All authors approved the final version of the report.

## Supplementary Material

Additional file 1 **Table S1**. BAC FISH results. Click here for file

Additional file 2 **Figure S1**. Array CGH profile of index case DNA Left panel) Whole chromosome X array profile. The scatter plot analysis shows a duplication in Xp22.2 (horizontal shift to the right of 0). Right panel) Zoomed-in gene view of left panel focussing on a 1.5 Mb window within Xp22.2 containing the duplication. Each point represents a single probe. Log_2 _(ratio) was plotted for all of oligonucleotide probes on the basis of their chromosome positions. The aberration calls identified by the ADM-2 algorithm (coloured areas) are shown. Click here for file

Additional file 3 **Figure S2**. X chromosome inactivation pattern. DXS6673E analysis. Electropherograms obtained from undigested and digested (HhaI and Rsa I enzymes) DNA of mother and patient. Both samples are heterozygous: patient genotype 1-3 (XCI ratio 82:18), mother genotype 1-2 (59:41). Click here for file

Additional file 4 **Table S2**. Chromatin immunoprecipitation assay results. Click here for file

Additional file 5 **Table S3**. Primers used for quantitative PCR. Click here for file

Additional file 6 **Table S4**. Primers used for ChIP and methylation assay. Click here for file
