# Supplementary material for: Juxtaposition of heterochromatic and euchromatic regions by chromosomal translocation mediates a heterochromatic long-range position effect associated with a severe neurological phenotype
Source: Mol Cytogenet. 2012 Apr 4;5:16. doi: 10.1186/1755-8166-5-16 (PMC3395859; doi:10.1186/1755-8166-5-16)
Supplement: Additional file 1 — Table S1. BAC FISH results. [file 1755-8166-5-16-S1.PDF]

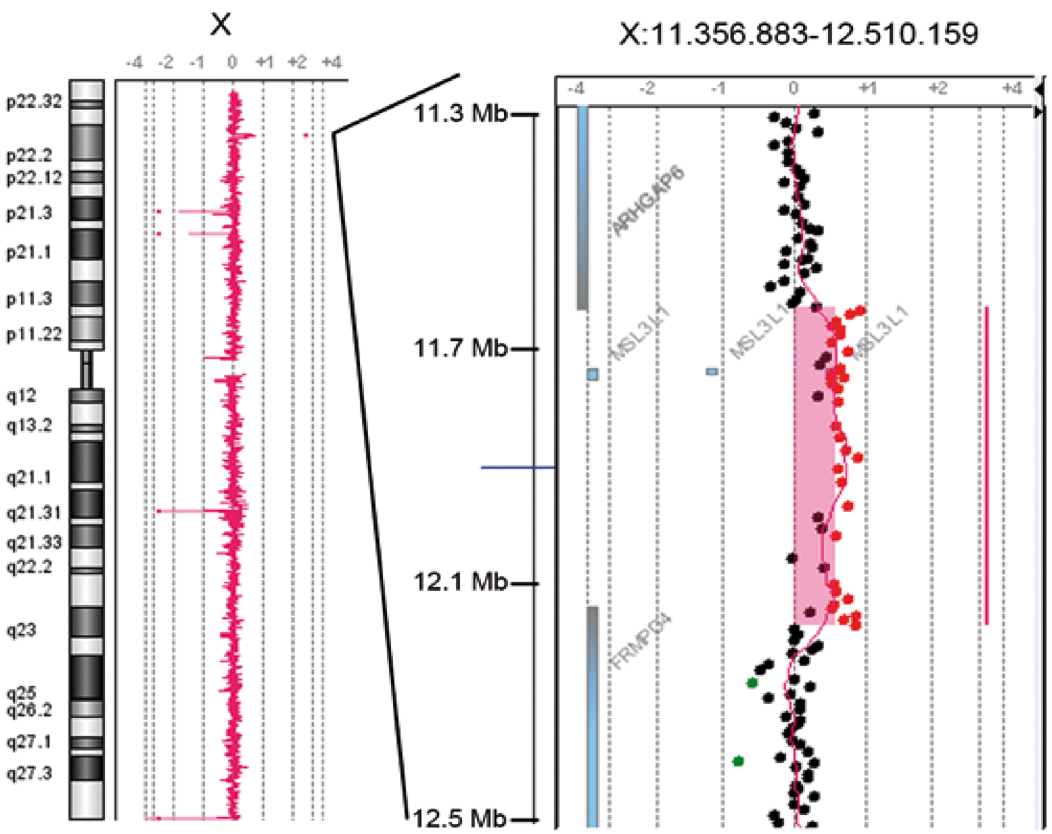

Supplementary Figure S1: Array CGH profile of index case DNA

Left panel) Whole chromosome X array profile. The scatter plot analysis shows a duplication in Xp22.2 (horizontal shift to the right of 0). Right panel) Zoomed-in gene view of left panel focussing on a 1.5 Mb window within Xp22.2 containing the duplication. Each point represents a single probe. Log_2 (ratio) was plotted for all of oligonucleotide probes on the basis of their chromosome positions. The aberration calls identified by the ADM-2 algorithm (coloured areas) are shown.
