# Supplementary material for: Juxtaposition of heterochromatic and euchromatic regions by chromosomal translocation mediates a heterochromatic long-range position effect associated with a severe neurological phenotype
Source: Mol Cytogenet. 2012 Apr 4;5:16. doi: 10.1186/1755-8166-5-16 (PMC3395859; doi:10.1186/1755-8166-5-16)
Supplement: Additional file 2 — Figure S1. Array CGH profile of index case DNA Left panel) Whole chromosome X array profile. The scatter plot analysis shows a duplication in Xp22.2 (horizontal shift to the right of 0). Right panel) Zoomed-in gene view of left panel focussing on a 1.5 Mb window within Xp22.2 containing the duplication. Each point represents a single probe. Log2 (ratio) was plotted for all of oligonucleotide probes on the basis of their chromosome positions. The aberration calls identified by the ADM-2 algorithm (coloured areas) are shown. [file 1755-8166-5-16-S2.PDF]

Table S1

BAC FISH results

Probe name	Chromosome localisation	FISH signal on derivative chromosomes	Position in release UCSC, hg19
RP11-696P19	16q11.2	der(16)	chr16:46,385,802-46,508,907
RP11-46D6	16q11.2	der(16)	chr16:46,501,304-46,671,860
RP11-91A22*	16q11.2	der(16)	chr16:46,520,735-46,692,088
RP11-283C7	16q12.1	der(16)	chr16:46,945,331-47,130,611
CTD-3056I17	16q12.1	der(16)	chr16:47,187,527-47,268,711
CTD-2177A24	16q12.1	der(16)+ der(15)*	chr16:47,224,031-47,287,723
RP11-671L23	16q12.1	der(16)+ der(15)	chr16:47,187,522-47,363,142
CTD-2027D24	16q12.1	der(16)+ der(15)	chr16:47,238,197-47,338,908
RP11-474B12	16q12.1	der(15)	chr16:47,316,866-47,469,918
RP11-152M8	16q12.1	der(15)	chr16:47,325,474-47,501,451
RP11-99P23	16q12.1	der(15)	chr16:47,465,093-47,629,829
RP11-245J16	16q12.1	der(15)	chr16:48,329,438-48,479,754
RP11-303G21	16q12.1	der(15)	chr16:48,438,656-48,439,328
RP11-462M23	16q12.1	der(15)	chr16:49,246,561-49,407,401
RP11-321D3	16q12.1	der(15)/15q°	chr16:50,275,355-50,464,909
RP11-20A10	16q12.1	der(15)	chr16:50,642,163-50,816,180
RP11-132F7	16q12.1	der(15)	chr16:52,416,039-52,585,245
RP11-497D8	16q12.1	der(15)	chr16:54,920,401-55,107,283
RP11-61I15	16q12.1	der(15)	hr16:55,264,498-55,427,318

Legend: *A very small hybridisation signal was observed on der(15).°The probe gave an additional signal on the 15q arm.
