# Supplementary figures and images for: Juxtaposition of heterochromatic and euchromatic regions by chromosomal translocation mediates a heterochromatic long-range position effect associated with a severe neurological phenotype
Source: Mol Cytogenet. 2012 Apr 4;5:16. doi: 10.1186/1755-8166-5-16 (PMC3395859; doi:10.1186/1755-8166-5-16)

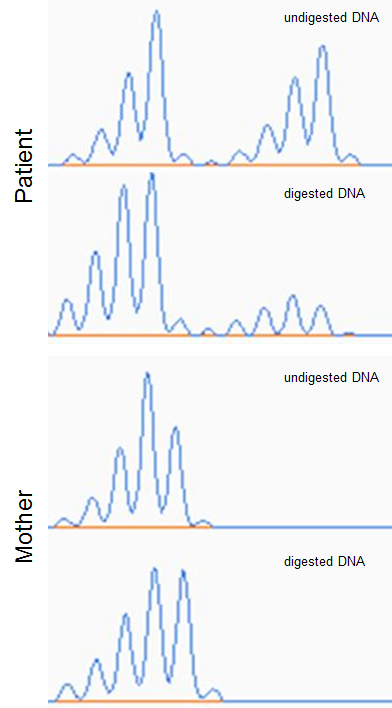

Supplement: Additional file 3 — Figure S2. X chromosome inactivation pattern. DXS6673E analysis. Electropherograms obtained from undigested and digested (HhaI and Rsa I enzymes) DNA of mother and patient. Both samples are heterozygous: patient genotype 1-3 (XCI ratio 82:18), mother genotype 1-2 (59:41). [file 1755-8166-5-16-S3.TIFF]
