# Supplementary material for: Juxtaposition of heterochromatic and euchromatic regions by chromosomal translocation mediates a heterochromatic long-range position effect associated with a severe neurological phenotype
Source: Mol Cytogenet. 2012 Apr 4;5:16. doi: 10.1186/1755-8166-5-16 (PMC3395859; doi:10.1186/1755-8166-5-16)
Supplement: Additional file 4 — Table S2. Chromatin immunoprecipitation assay results. [file 1755-8166-5-16-S4.PDF]

## Table S2

Chromatin immunoprecipitation assay results

| | Primers | t(15;16) patient | | | | | | Normal control | | | | | |
|---|---|---|---|---|---|---|---|---|---|---|---|---|---|
| | | AcH3 | AcH4 | H3Dim and TrimK4 | H3Dim K9 | H3Trim K9 | H3Dim K27 | AcH3 | AcH4 | H3Dim and TrimK4 | H3Dim K9 | H3Trim K9 | H3Dim K27 |
| **VPS35** | | + | + | + | - | - | nd | + | + | + | - | - | nd |
| | | | | | | | | | | | | | |
| **NETO2** | 1 | + | + | + | + | - | + | + | + | + | - | - | + |
| | 2 | + | + | + | + | + | + | + | + | + | - | - | - |
| | | | | | | | | | | | | | |
| **SIAH1** | 1 | + | + | + | - | - | - | + | + | + | - | - | - |
| | 2 | + | + | + | - | - | - | + | + | + | - | - | - |
| | | | | | | | | | | | | | |
| **PGK1** | | + | + | + | + | + | + | + | + | + | + | + | + |

For all assays, chromatin before immunoprecipitation was used as a positive control and the experiment with no antibody as a negative control. The primers sequences are listed in Supplementary Table S4; nd = not determined.

The PGK1 promoter was used as an immunoprecipitation control because it shows all types of analysed chromatin modifications.[37]
