# Supplementary material for: Juxtaposition of heterochromatic and euchromatic regions by chromosomal translocation mediates a heterochromatic long-range position effect associated with a severe neurological phenotype
Source: Mol Cytogenet. 2012 Apr 4;5:16. doi: 10.1186/1755-8166-5-16 (PMC3395859; doi:10.1186/1755-8166-5-16)
Supplement: Additional file 5 — Table S3. Primers used for quantitative PCR. [file 1755-8166-5-16-S5.PDF]

On-line Supplementary Table S3

Gene		Primers used for quantitative PCR
VPS35	1	fw: gggacatagccgagatagagaaaa; rev: ctgaggcgcaccaaatttg
	2	fw: attcatctgctgcgctctga; rev: acattttggagctggtggaaa
ORC6L	1	fw: agacacagcaagtggatcttgact; rev: agaatcttgcatgctgaaagca
NETO2	1	fw: ggcagacttgctggaagaattg; rev: gtggtggtcgtggatgca
ITFG1	1	fw: gttgctgttcatccacaaatgg ; rev: agcagtgggttccagtcctaca
	2	fw: aggtcggagcgcaaatttt; rev: tgttttcgtatagatttttctccagatg
ABCC12	1	fw: gaccccagcctgaagaccat; rev: gtagcccggcatcatcca
	2	fw: aaagccccccatcttacatca ; rev: ccatgtcaaggtggcatttg
	3	fw: ttgtgaaaagggaacccacaag ; rev: atcctcgcaggttgtgaatca
SIAH1	1	fw: ccaacaatgacttggcgagtct; rev: tggccactctgacattgaagaa
	2	fw: tgtgttaccgcccattcttca; rev: tggacaacatgtgagctttgg
	3	fw: gaacacgcaagcaagctgaa; rev: gtcgcttcccaagtcaatcg
CBLN1	1	fw: gatgagtaatcgcaccatgatca; rev: agtgctgcgttctgaatcaaag
	2	fw: gaaaggcgaccgagcatacc ; rev: ggaatccggagaaggtcgagta
ADCY7	1	fw: atgagctctttggcaagttcga ; rev: gtcgccgaggatcttgattc
	2	fw: gctgtgtcctgggcttcatc ; rev: actgtcagcagcacaacctttg
BRD7	1	fw: agcttgaccgcatcgtgaag ; rev: aaattcgcactgactgttcacaa
	2	fw: aaaagaaatggagcagattacagaagt ; rev: gcgctatgagcctgtgttgag
CYLD	1	fw: ttgccaggaaaagaaagcttagg ; rev: ccatcccagttgccaatagg
	2	fw: agagtgtgacgcaggaaagga ; rev: gatgaacctttgtccccaaca
SALL1	1	fw: catcatctgtcaccgggttct ; rev: tctccccagtgtgtgtcctgta
	2	fw: aacgggctggcgatgaa ; rev: cgaggcttccaggaattgg
RBL2	2	fw: ccgcagcatgagcgaaa ; rev: aagccacatataaggcacatgcta
	3	fw: acaatgggcaaacggtaacc ; rev: gcctgccccccaacat
HERPUD1	1	fw: cgttgggtggtttccatttaga ; rev: caacgtcaggaggaggaccat
	2	fw: cccctggatgggaaaacat ; rev: ggtgtgtaaccggagaaacca
DNAJA2	1	fw: gccagtttaggccccactagt ; rev: aagaaggccaaggaaggaaaa
	2	fw: ggaccactaccccttatcgtaag; rev: agaaagtttctacaggagacacacctt
SHCBP1	1	fw: aggtgctggttgagatcacaga ; rev: agtcacagaggtatggttcagcaa
	2	fw: tggtaagactacgctggaaaactg ; rev: tctgctgatgtccgcactgt
GPT2	1	fw: ctgacggaagacctgtttaaccaa ; rev: atccgagggaaggcgtacat
	2	fw: cagagaagctcgttccatgaaa ; rev: tcaagctgcataatcctctgaaaa
CKLF		fw: tgaaaggccacgtgaagatg ; rev: ttgtgcgatgataaaaaaggtcat
HPRT		fw: gcttgctggtgaaaaggacc; rev: gtcaagggcatatcctacaac
