# Supplementary material for: Juxtaposition of heterochromatic and euchromatic regions by chromosomal translocation mediates a heterochromatic long-range position effect associated with a severe neurological phenotype
Source: Mol Cytogenet. 2012 Apr 4;5:16. doi: 10.1186/1755-8166-5-16 (PMC3395859; doi:10.1186/1755-8166-5-16)
Supplement: Additional file 6 — Table S4. Primers used for ChIP and methylation assay. [file 1755-8166-5-16-S6.DOC]

**Table S4**

Primers used for ChIP and methylation assay

| **Gene** | **Primers used for DNA methylation and ChIP assay** | | **Amplicon localisation (hg19)** |
| --- | --- | --- | --- |
|  |  |  |  |
| *VPS35* | 1 | fw: 5' aagtaggccaatcaatgagc 3' and rev: 5'agcaggggctacaaggag 3' | **chr16:46,722,989-46,723,187** |
|  |  |  |  |
| *NETO2* | 1 | fw: 5' gcaggacctgagctctcc 3' and rev: 5' ctttgaggaccgagcaga 3' | **chr16:47,177,518-47,177,647** |
|  | 2 | fw: 5' gagaggcaccctcactacc and rev: 5'gcccaggctcatttaagg 3' | **chr16:47,177,898-47,178,037** |
|  | 3* | fw: 5'agtttattgtggggttygggtatg 3' and rev bio5' caaaaccrctccaaaaccatatt 3'seq 5'gggaggtagttgttgg 3' | **chr16:47,177,504-47,177,707**  **chr16:47,177,633-47,177,646** |
|  |
|  |  |  |  |
| *SHA1* | 1 | fw: 5' gcgctctcgagaggcggcg 3' and rev: 5' acttacctgtgggcggagagc 3' | **chr16:48,419,130-48,419,224** |
|  | 2 | fw: 5' agcaacggtagccgagta 3' and rev: 5' cgctaaataccacacactgc 3' | **chr16:48,418,536-48,419,342** |
|  | 3* | fw: 5 'gygggaggagttgttttg 3' and rev bio5' ccccrctaaataccacacact 3'  seq 5' ggaggagttgttttgag 3' | **chr16:48,419,340-48,419,632**  **chr16:48,419,614-48,419,628** |
|  |
|  |  |  |  |
| *CYLD* | 1 | fw: 5' ttaacccagcccaaacctaa 3' and rev: 5' ggactttccgctctgtcaag | **chr16:50,775,581-50,775,816** |
|  | 2 | fw: 5' cccaccccatttacagattg 3' and rev: 5' ccctagaaagggggaaactg | **chr16:50,776,461-50,776,689** |
|  | 3* | fw: 5’tggaggaaggtttgttataggga 3’ rev: 5’ caaccaaacctactacctaaaccc 3’  seq: 5’ ttgttatagggaggtttaat 3’ | **chr16:50,775,848-50,776,011 chr16:50,775,856-50,775,875** |
|  | 4* | fw: 5’gggatttttagaggagagaggat 3’ rev: 5’ cctatacctcaatctcccaatcta 3’ seq: 5’ gaggagagaggatttgg 3’ | **chr16:50,776,397-50,776,497 chr16:50,776,407-50,776,422** |
|  |  |  |  |
| *RBL2* | 1 | fw: 5' tcgtcagtacagccctgttg 3' and rev: 5' caggcacccgtagtcttgag 3' | **chr16:53,468,149-53,468,338** |
|  | 2 | fw: 5' cagcccgtgctcaaga 3' and rev: 5' gtcgtcctcctcctcctcat 3' | **chr16:53,468,310-53,468,552** |
|  | 3 | fw: 5' ttgaggaaaatgggtgtgtg 3' and rev: 5' gttaacagggggaagcgaac 3' | **chr16:53,468,973-53,469,157** |
|  | 4* | Fw: 5’ggggtttcgttgaggaaaat 3’ and rev: 5’ caacccaaaacgcatcaacac 3’  Seq: 5’ gggatttgtttttatttgta 3’ | **chr16:53,468,964-53,469,219**  **chr16:53,469,070-53,469,089** |
|  |  |  |  |
| *PGK1* | 1* | See [ref](http://www.ncbi.nlm.nih.gov/pubmed/15781624?ordinalpos=3&itool=EntrezSystem2.PEntrez.Pubmed.Pubmed_ResultsPanel.Pubmed_RVDocSum) 41 |  |
|  |  |  |  |
| *HPRT* | 1 | fw: 5’gcttgctggtgaaaaggacc 3’ and rev 5’gtcaagggcatatcctacaac 3’ |  |
|  |  |  |  |

Legend: *Primers used in the pyrosequencing experiments.
